# MYC_V1-Related Genes Affect Gastric Cancer Proliferation by Regulating Energy Metabolism and Analysis of Therapeutic Targets

**DOI:** 10.3390/ijms27114862

**Published:** 2026-05-28

**Authors:** Duo Xu, Lingyi Peng, Jing He, Xue Wang, Jiaqi Xia

**Affiliations:** 1 School of Clinical Medical Sciences, Jiamusi University, Jiamusi 154007, China; wqqv54@163.com (D.X.); 22110150628@stu.jmsu.edu.cn (L.P.); 2 School of Basic Medical Sciences, Jiamusi University, Jiamusi 154007, China; 228163011@stu.jmsu.edu.cn; 3 School of Public Health, Jiamusi University, Jiamusi 154007, China; xuewang1025@163.com

**Keywords:** gastric cancer, energy metabolism, prognosis model, MYC_V1

## Abstract

Gastric cancer (GC) is the fifth leading cause of cancer-related mortality worldwide. Treatment options for advanced GC remain limited, owing to the frequent emergence of drug resistance. This highlights an urgent clinical need for novel therapeutic targets. Abnormal energy metabolism is a hallmark feature of cancer. MYC_V1-driven metabolic reprogramming plays a pivotal role in tumor progression. However, the specific mechanisms by which MYC_V1-related genes regulate energy metabolism in GC remains poorly understood. We employed single-sample gene set enrichment analysis (ssGSEA) to evaluate multiple tumor hallmarks in GC. A prognostic risk model was constructed based on MYC_V1-related genes, with the risk score (RS) used to stratify patients into distinct risk groups. A nomogram was developed and validated using calibration curves. Through the systematic molecular docking screening of 8327 compounds, potential therapeutic agents were identified. Functional experiments, including the CCK-8 assay, wound-healing assay and ATP production assay, were conducted to validate the role of *NDUFV2* in GC progression. This study identified MYC_V1 as the primary risk factor affecting the overall survival (OS) in GC patients (*p* = 0.038). A prognostic risk model was successfully constructed based on eight MYC_V1-related genes (*KPNA2*, *MCM2*, *MCM4*, *NDUFV2*, *PDK4*, *MPO*, *IGFBP1*, and *STC2*). The RS was confirmed as an independent prognostic factor. The prognostic risk model accurately predicted patient 1-, 3-, and 5-year OS in GC patients. Tumor microenvironment analysis revealed significant differences in immune cell infiltration patterns between high-risk and low-risk groups. High-throughput drug screening and molecular docking identified camptothecin (CPT) and vinblastine as showing strong therapeutic potential for high-risk patients. Experimental validation demonstrated that *NDUFV2* was significantly overexpressed in GC tissues, and its knockdown markedly suppressed the proliferation, migration capacity, and intracellular ATP production in GC cells, confirming the critical role of *NDUFV2* in GC progression. These findings establish *NDUFV2* as a potential therapeutic target in GC.

## 1. Introduction

GC is a malignant tumor of the digestive tract: it is an extremely aggressive tumor, and it remains the fifth leading cause of cancer-related death worldwide [1,2,3]. The poor prognosis of GC patients and the problem of drug resistance in advanced treatment have always been difficult problems in clinical diagnosis and treatment [4,5,6], seriously compromising patient survival and quality of life [3,5]. The efficacy of current chemotherapy and targeted therapies is often limited by intrinsic or acquired resistance, highlighting an urgent need for novel therapeutic targets [7]. Metabolic abnormalities associated with GC arise from disrupted energy and material metabolism during GC cell growth, primarily manifesting as abnormalities in metabolic pathways involving carbohydrates, lipids, amino acids, and other substance [8,9]. The identification of reliable biomarkers derived from metabolic pathway analysis will significantly advance precision cancer stratification and enable standardized treatment protocols for GC. Consequently, there is a compelling clinical need to identify novel biomarkers and effective therapeutic targets that can better select GC patients for optimal combination therapies.

Metabolic reprogramming has attracted much attention as a hallmark feature of tumors [10,11,12,13]. Abnormal energy metabolism provides both the material basis and energetic support for tumor initiation and progression, and has become an attractive target for therapeutic intervention [3,5]. The transcription factor MYC drives tumor metabolism by directly orchestrating genes that control nutrient uptake and utilization. MYC regulates target genes involved in various pathways such as cell proliferation, metabolism, and immune evasion, playing a critical role in the tumor initiation and development in multiple types of cancer [14,15,16,17]. Because of its high prevalence of deregulation and its causal role in cancer formation, maintenance, and progression, targeting *MYC* is theoretically an attractive strategy for treating cancer [18]. Studies have demonstrated that *MYC* pathway alterations are associated with aggressive clinical features in hepatocellular carcinoma [19]. Thus, *MYC* may represent a core therapeutic target in GC [20]. The downregulation of *MYC* plays a tumor suppressor role in GC development [21]. This makes MYC an appealing target, despite it being previously considered an undruggable protein [22]. And we found that proteins in the MYC pathway may reduce the proliferation, invasion, and migration abilities of GC cells by downregulating *MYC*. The oncogenic functions of MYC involve the regulation of gene networks that coordinate cell proliferation and metabolic processes [23]. In this study, we defined a gene signature named MYC_V1, which was derived from MYC pathway-related genes using ssGSEA. MYC_V1 represents the overall activity of MYC-driven transcriptional programs and is not a specific transcript variant of the MYC gene.

MYC transcriptionally upregulates *NDUFV2* expression, thereby enhancing mitochondrial Complex I activity and promoting cancer cell proliferation. The *NDUFV2* gene participates in mitochondrial respiration, encoding NDUFV2, which is the core subunit of the mitochondrial membrane respiratory chain NADH dehydrogenase (Complex I) [24,25,26]. Notably, *NDUFV2* is highly expressed in prostate cancer as an unfavorable prognostic marker, and its silencing inhibits the proliferation of drug-resistant breast and liver cancer cells [27,28]. However, the role and intrinsic relationship of abnormal *NDUFV2* expression in the development and progression of GC have not yet been fully elucidated and require further in-depth investigation.

Currently, the specific mechanism by which MYC_V1 regulates energy metabolism in GC within core energy metabolism pathways remains to be investigated [29,30,31,32,33,34]. The research on screening anticancer drugs targeting the MYC_V1 energy metabolism axis is relatively limited, which hinders the development of precise treatment strategies for GC. Therefore, elucidating how MYC_V1 drives GC progression via metabolic reprogramming and identifying potential anticancer agents targeting this axis are of paramount importance [35,36]. This research is significant for overcoming current treatment challenges and enhancing the effectiveness of GC treatments, potentially opening new avenues for precise GC therapy [37,38]. We hypothesize that the transcription factor MYC drives metabolic reprogramming, in part, by directly transactivating the expression of *NDUFV2*—a core subunit of mitochondrial complex I. This MYC/NDUFV2 axis is proposed to enhance complex I activity, thereby boosting oxidative phosphorylation to fuel GC cell proliferation, invasion, and metabolic adaptation. Furthermore, this study will assess the effect of NDUFV2 on mitochondrial energy metabolism by measuring ATP production. Recent studies have identified various prognostic biomarkers in GC. These markers facilitate patient stratification and guide treatment decisions. Metabolic genes have emerged as promising candidates for targeted therapy. Drug sensitivity prediction based on genomic profiles enables personalized treatment strategies. The integration of prognostic and predictive biomarkers represents a key approach for improving GC outcomes.

## 2. Results

### 2.1. MYC_V1 Represents a Primary Risk Factor for Survival in GC Patients

**Figure 1:**
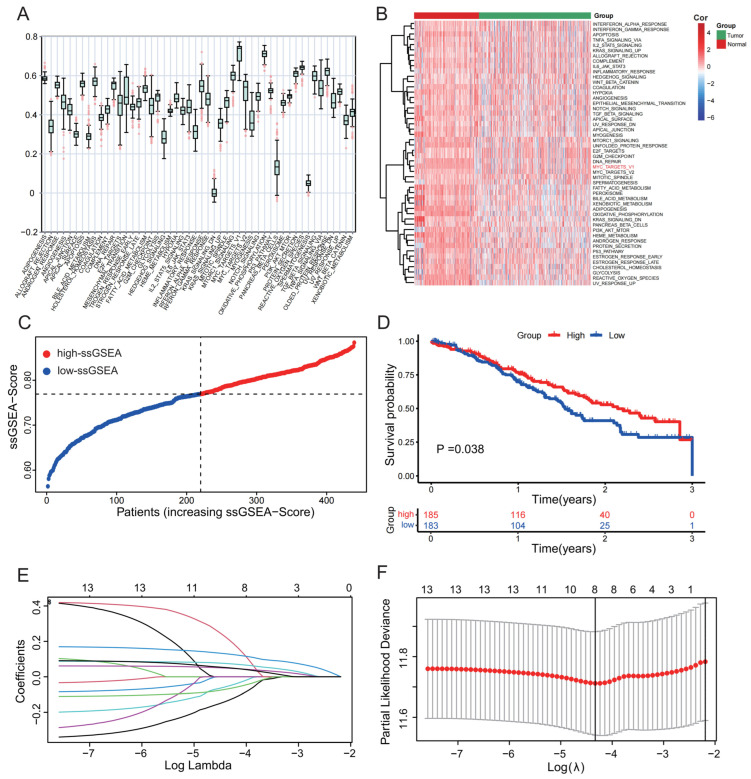
MYC_V1-related genes is identified as the primary risk factor for survival. (**A**) Univariate Cox regression analysis indicated that MYC_V1-related genes were the primary risk factor among various hallmarks of cancer (50 pathways). (**B**) Heatmap showing differentially enriched biological pathways between normal and GC tumor tissues (N = 36, T = 384). (**C**,**D**) Kaplan–Meier analysis showed that patients with higher ssGSEA scores displayed worse OS. Survival difference was compared using the log-rank test. (**E**) LASSO coefficient profiles of the 8 genes. Each curve corresponds to a gene. (**F**) Partial likelihood deviance for the LASSO coefficient profiles.

We estimated and ranked the Cox coefficients for each cancer hallmark. Among them, MYC_V1, which is closely associated with energy metabolism, was aberrantly hyperactivated in GC (Figure 1A,B). Next, we divided GC patients into two groups based on the median Z-score and found that patients with lower Z-scores had significantly longer overall survival (OS) (Figure 1C,D). These findings indicate that MYC_V1 is a strong prognostic factor associated with OS in GC patients. To construct a prognostic risk model based on MYC_V1-related energy metabolism genes, we performed LASSO-Cox analysis on the 13 genes that are the intersection of the MYC-pathway-related genes and the differentially expressed genes. According to the optimal λ value (indicated by the vertical dashed line on the left based on the minimum standardized value), we selected eight genes to construct the prognostic model (Figure 1E,F). The optimal λ value was determined by 10-fold cross-validation based on the minimum partial likelihood deviance. At this λ value, the LASSO model retained eight genes with non-zero coefficients, which were used to construct the final prognostic risk model. The resulting optimal risk score (RS) model formula was as follows: RS = −0.051101155 × (expression of *KPNA2*) − 0.131031318 × (expression of *MCM2*) + 0.208856439668148 × (expression of *MCM4*) − 0.067071073 × (expression of *NDUFV2*) + 0.128098124167172 × (expression of *PDK4*) + 0.0739124272667695 × (expression of *MPO*) + 0.0483205448342285 × (expression of *IGFBP1*) + 0.0504939011675305 × (expression of *STC2*). It should be noted that the prognostic risk model described above was constructed primarily based on the descriptive analysis of transcriptomic data, aiming to provide a risk stratification tool for gastric cancer patients. The functional regulatory relationships of the genes in this model are validated in subsequent in vitro experiments. Analysis using the GEPIA2 database revealed that *MYC* and the eight key genes were closely associated with favorable disease-free survival in GC patients. (Figure S1). Based on the genomic analysis of GC patients in the TCGA cohort, we examined the mutation landscape associated with the prognostic gene signature. The eight genes comprising the signature (*MCM2*, *MCM4*, *NDUFV2*, *PDK4*, *IGFBP1*, *KPNA2*, *MPO*, and *STC2*) exhibited varying degrees of genetic alteration, with copy number variation frequencies ranging from 1% to 3% across the cohort. In addition, we summarized the top 10 most frequently mutated genes in GC (Figure S2A–C). The copy number variation (CNV) analysis of the prognostic signature genes revealed that all eight prognostic genes exhibited both copy number amplification and deletion events, with the amplification frequency (GAIN) generally being higher than the deletion frequency (LOSS). These genes are distributed across distinct chromosomal regions (Figure S2D,E). We also analyzed the role of the aforementioned genes in metabolic pathways (Figure S3). The key genes identified in this study play important roles in GC. The results described above demonstrate that the MYC_V1-related energy metabolism genes identified in this study play important roles in gastric cancer.

### 2.2. Evaluation Prognostic Risk Model and External Validation

**Figure 2:**
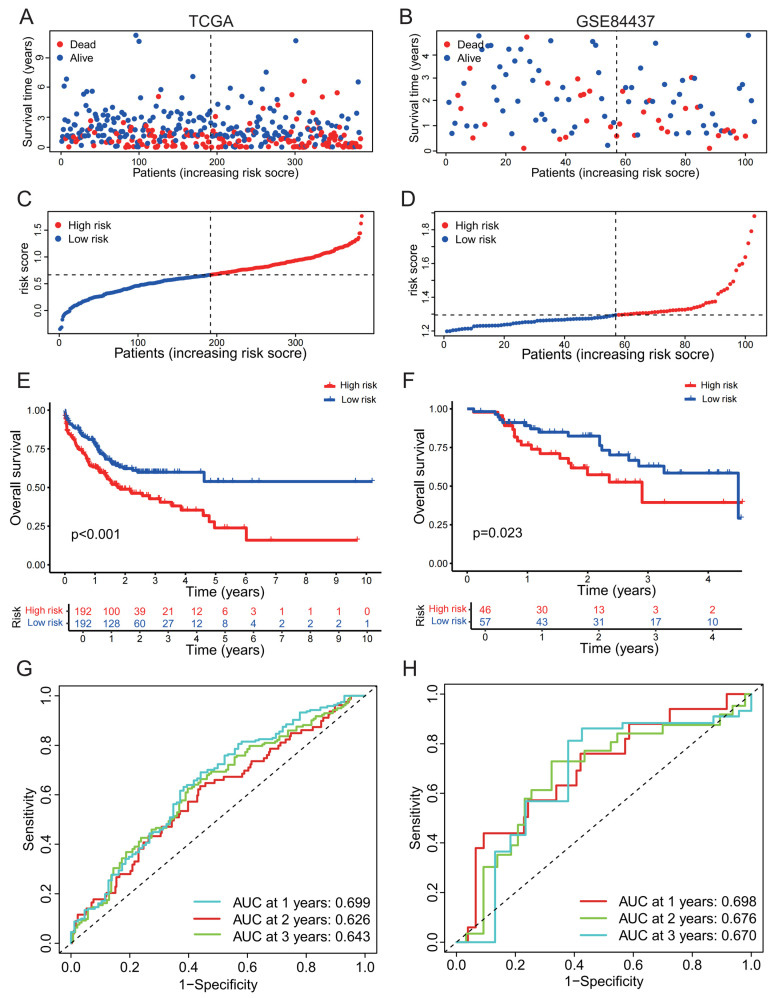
Predictive value of MYC_V1-related genes RS prognostic model in the training and test group. (**A**) Risk score distribution in the TCGA cohort. (**B**) Risk score distribution in the GSE84437 cohort. (**C**,**D**) Scatterplots of patients with different survival status and survival time. (**E**,**F**) Kaplan–Meier survival curves of OS in different risk groups. (**G**,**H**) Time-dependent ROC curves for OS prediction at 1, 2, and 3 years in the TCGA cohort (**G**) and the GSE84437 cohort (**H**).

To demonstrate the reliability of MYC_V1-related energy metabolism prognostic risk model, we divided the 384 patients from the TCGA-STAD cohort in the training set into a high-risk group (n = 192) and a low-risk group (n = 192) based on the median RS threshold (Figure 2A). Compared with the low-risk group, the high-risk group had a higher mortality rate and shorter survival time. The number of deaths increased with increasing RS (Figure 2C). Kaplan-Meier (KM) survival curve analysis indicated a significantly better prognosis for patients with lower RS (Figure 2E). Time-dependent ROC analysis revealed the prognostic accuracy of the model for OS (Figure 2G). To validate the accuracy of the model, we extracted 116 GC patients from the GSE84437 external validation cohort and calculated their RS using the same formula as the training set. Based on the median RS value, 58 patients were categorized into the low-risk group and 58 into the high-risk group (Figure 2B). Patients in the low-risk group had longer survival times than those in the high-risk group (Figure 2D). Furthermore, KM analysis disclosed a significant discrepancy in survival rates between the low-risk and high-risk groups (*p* < 0.05, Figure 2F), which was consistent with the results obtained from the training set. To further validate the prognostic performance of the risk model in an independent cohort, we performed time-dependent ROC analysis on the GSE84437 dataset (Figure 2H).

### 2.3. Independent Prognostic Analysis of Risk Score and Clinical Characterstics

**Figure 3:**
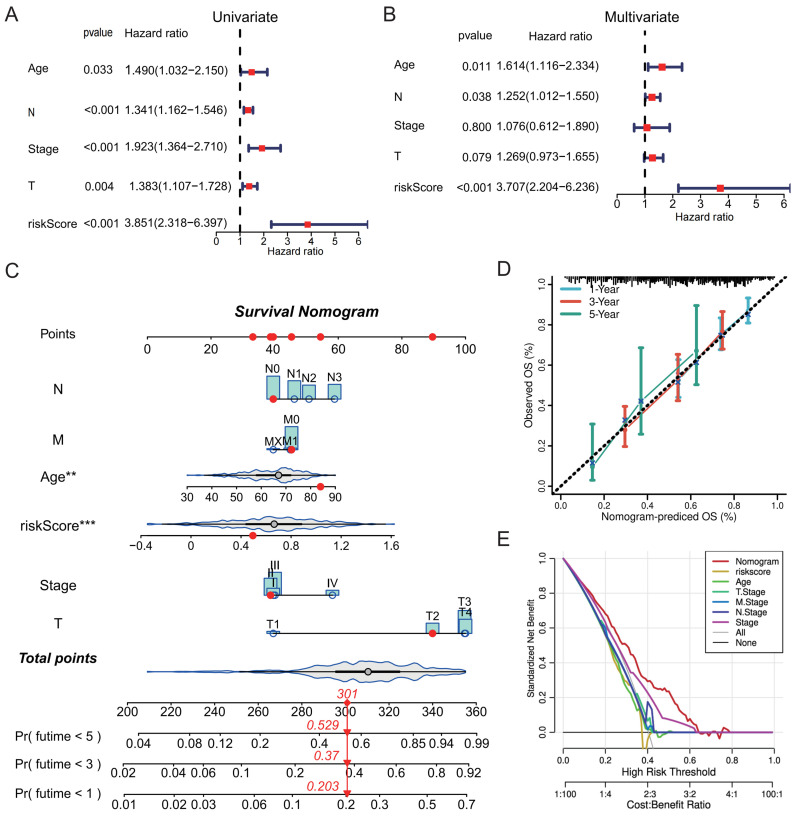
Construction and validation of nomogram for predicting survival probability of GC. (**A**,**B**) Univariate and multivariate Cox regression analyses of the MYC_V1-related genes RS and other clinical features to screen for factors independently associated with prognosis. (**C**) Nomogram combining RS with pathologic features. ** *p* < 0.01, and *** *p* < 0.001 (**D**) Calibration plots for predicting 1-, 3-, and 5-year OS of patients. (**E**) Concordance index (C-index) was generated to assess the identification and forecasting capabilities of the nomogram.

To verify whether the RS could serve as an independent prognostic factor, we performed univariate and multivariate Cox regression analyses on the RS and other clinical features including age and TNM stage. The RS was independently correlated with OS in GC patients, which suggested that they may act as independent prognostic predictors and have a better prognosis than clinical indicators (*p* < 0.001) (Figure 3A,B). Afterwards, a nomogram was constructed to derive a score for each factor based on patient-specific information and ultimately aiming to predict the patient’s OS probability based on the total score (Figure 3C). According to the nomogram, the RS was an exceedingly vital factor among various clinical parameters. We generated calibration curves to assess the accuracy of the nomogram in predicting the actual survival outcomes of patients with GC, demonstrating its high concordance with the actual survival of patients (Figure 3D). Furthermore, the prognostic risk model and TNM stage (the TNM stage refers to the tumor-node-metastasis classification system according to the American Joint Committee on Cancer) had a high differentiating capacity, according to the concordance index (C-index) (Figure 3E). To assess the association of the MYC_V1-based risk score with clinicopathological features in GC, we performed correlation analyses. The risk score showed no significant link to patient age (*p* = 0.49) but was strongly associated with survival status, being markedly higher in deceased patients (*p* = 2.3 × 10^−7^). It also varied significantly across TNM stages (*p* = 0.028) and increased with lymph node metastasis severity (*p* = 0.012) (Figure S4).

### 2.4. Analysis of GC Immune Microenvironment

**Figure 4:**
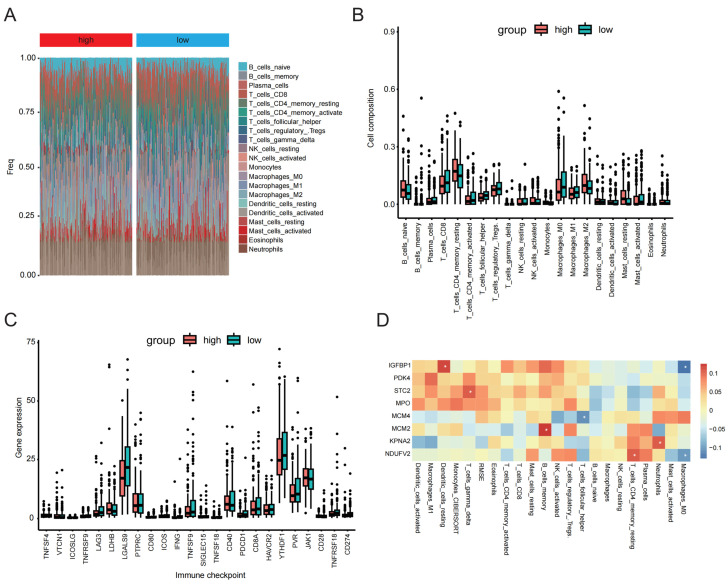
Tumor microenvironment and immune cell infiltration. (**A**) Proportion of 21 immune cells between high- and low-risk groups. (**B**) Relative proportion of immune cell infiltration in high-risk and low-risk group. Green represents the low-risk group. Red represents the high-risk group. (**C**) Comparison of the expression differences of immune-checkpoint-related genes between high- and low-risk group. (**D**) Heatmap of the correlations between MYC_V1-related genes (*IGFBP1*, *STC2*, *KPNA2*, *MCM2*, *MCM4*, *NDUFV2*, *PDK4*, and *MPO*) and 21 immune cells, * represent *p* < 0.05.

We conducted an enrichment analysis on the differentially expressed genes between the high-risk and low-risk groups (Figure 4A). We found that Macrophages M0 and NK cells were significantly enriched in low-risk patients (Figure 4B). Subsequently, by comparing the expression differences of immune-checkpoint-related genes between the high-risk and low-risk groups, further insights can be gained into the immune escape mechanisms of GC (Figure 4C). As shown in Figure 4D, we present the relationships between these genes and the infiltration levels of 21 different immune cell types in the form of a heatmap. Notably, there was a significant positive correlation between IGFBP1, PDK4, and NK cells, suggesting potential roles for these genes in modulating the GC tumor immune microenvironment.

### 2.5. NDUFV2 Is Overexpressed in GC and Promotes Tumor Cell Proliferation and Migration

**Figure 5:**
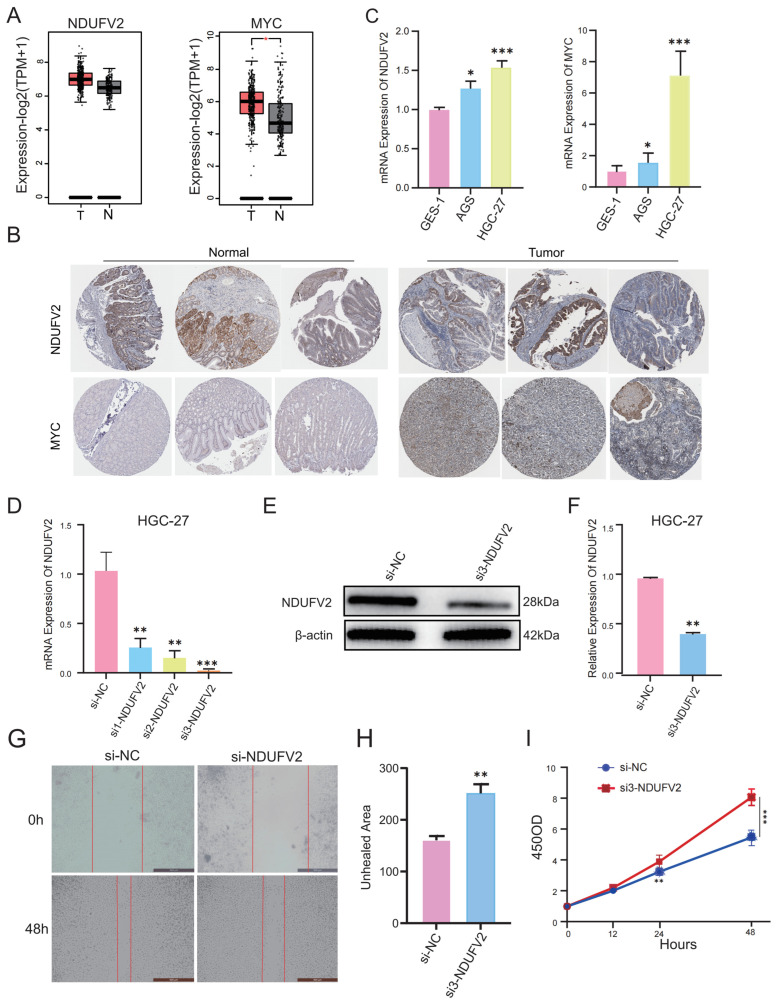
*NDUFV2* is highly expressed in GC and promotes malignant phenotypes. (**A**) Scatterplot shows coordinated high mRNA expression of *MYC* and *NDUFV2* in GC tissues compared to normal tissues. (**B**) IHC images show NDUFV2 and MYC protein expression in GC. (**C**) mRNA expression levels of *NDUFV2* and *MYC* in the normal gastric epithelial cell line GES-1 and GC cell lines AGS and HGC-27. (**D**) Validation of *NDUFV2* knockdown efficiency at the mRNA level. (**E**) Western blot analysis NDUFV2 protein expression, (**F**) and corresponding quantitative bar graph, ** *p* < 0.01. (**G**,**H**) Wound-healing assay assessing the migratory capacity of HGC-27 cells upon *NDUFV2* knockdown. (**I**) CCK-8 assay evaluating cell proliferation following *NDUFV2* knockdown. OD values were monitored at indicated time points (0, 12, 24, and 48 h). Data of gene expression are the means ± SEM of three experiments, each performed in duplicate. * *p* < 0.05, ** *p* < 0.01, and *** *p* < 0.001 vs. Control with Student’s *t* test (transport data) or one-sample *t* test (data of gene expression).

To investigate the role of *NDUFV2* in GC, we first analyzed its expression pattern alongside the oncogene *MYC*. The bioinformatic analysis of a public transcriptomic dataset revealed that both *NDUFV2* and *MYC* mRNA levels showed an upregulation trend in gastric tumor tissues compared to their normal counterparts (Figure 5A). Immunohistochemistry images from the HPA database confirmed these findings at the protein level. Clinical GC specimens exhibited strong positive staining for both NDUFV2 and MYC (Figure 5B). To identify genes potentially co-expressed with *NDUFV2* in GC, we performed a correlation analysis using the GEPIA2 database. *NDUFV2* expression was positively correlated with *HSD17B10*, *PSMG2*, and *VAPA* expression levels (Figure S5A–C). Quantitative PCR assays confirmed the *NDUFV2* overexpression in GC cell lines. *NDUFV2* and *MYC* mRNA levels were markedly higher in AGS and HGC-27 cells than in the normal gastric mucosal cell line GES-1 (Figure 5C). To delineate the functional impact of *NDUFV2* upregulation, we silenced its expression in HGC-27 cells using siRNA. Successful knockdown was verified at both the mRNA and protein levels (Figure 5D–F). *NDUFV2* mRNA levels were reduced by approximately 90% (*p* < 0.001) and protein levels by approximately 85% compared to the si-NC group. The si3-*NDUFV2* target demonstrated the most potent knockdown efficacy and was selected for further investigation. To determine whether *NDUFV2* knockdown affects the expression of its co-expressed genes, we measured their mRNA levels after silencing. *HSD17B10*, *PSMG2*, and *VAPA* mRNA levels were significantly downregulated following *NDUFV2* knockdown (Figure S5D). These results suggest that *NDUFV2* may act as an upstream regulator of these genes. Functional assays were performed to assess the impact of *NDUFV2* knockdown on GC cell behavior. Wound-healing assays showed that *NDUFV2* knockdown markedly reduced cancer wound closure (Figure 5G,H). Compared to the si-NC group, the unhealed area in si-NDUFV2 was increased by approximately 50%. CCK-8 assays demonstrated that *NDUFV2* knockdown potently inhibited cell proliferation. *NDUFV2* knockdown reduced cell viability by approximately 40% at 48 h (OD450: 1.20 ± 0.05 vs. 0.72 ± 0.04, *p* < 0.001). (Figure 5I). Collectively, these results indicate that NDUFV2 is overexpressed in GC and plays a crucial role in driving tumor cell proliferation and migration.

### 2.6. Based on the MYC_V1/NDUFV2 Axis, Camptothecin Combined with NDUFV2 Knockdown Synergistically Suppressed GC Cell Proliferation and Migration

**Figure 6:**
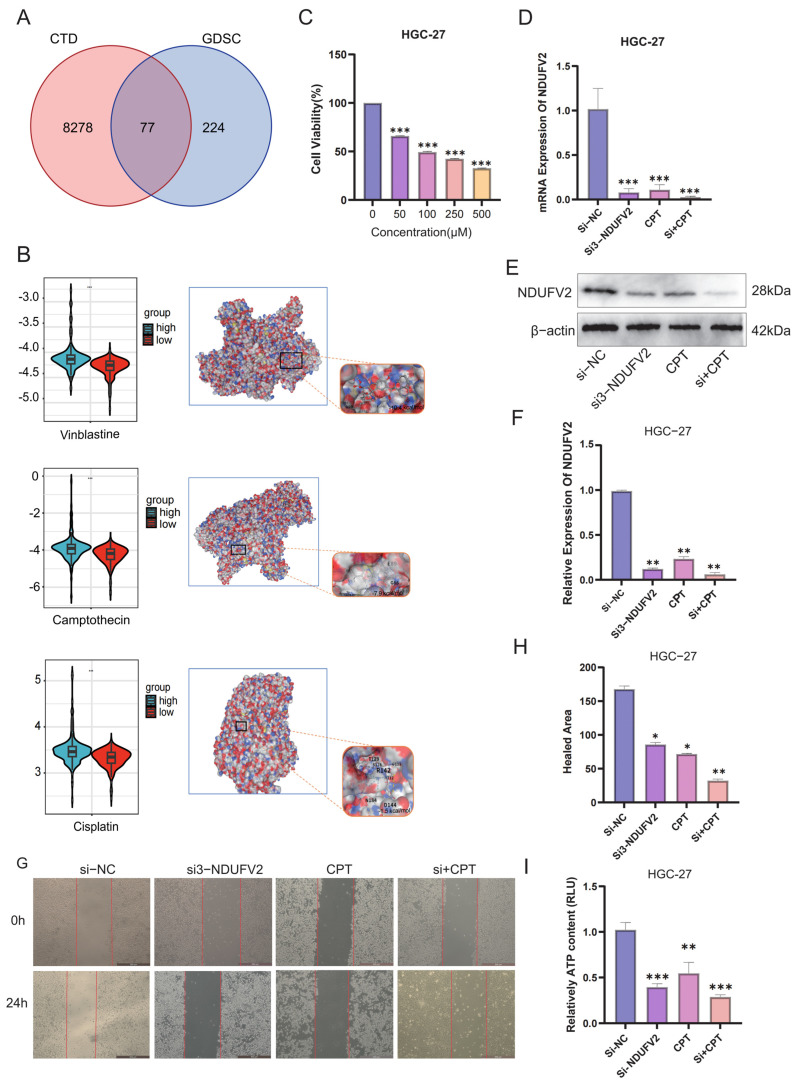
*NDUFV2* knockdown potentiates CPT in GC. (**A**) Venn diagram of drug screening. (**B**) Comparison of predicted IC50 values for core drugs between high-risk and low-risk GC patient groups, along with molecular docking results. (**C**) CCK-8 assay for IC50 determination. (**D**) Detection of *NDUFV2* mRNA expression levels by qRT-PCR. (**E**) Western blot analysis of NDUFV2 protein expression. (**F**) and corresponding quantitative bar graph. (**G**,**H**) Wound-healing assay assessing the migratory capacity of HGC-27 cells upon *NDUFV2* knockdown. (**I**) ATP production assay in HGC-27 cells following different treatments. Data of gene expression are the means ± SEM of three experiments, each performed in duplicate. * *p* < 0.05, ** *p* < 0.01, and *** *p* < 0.001 vs. Control with Student’s *t* test (transport data) or one-sample *t* test (data of gene expression).

Based on the potential therapeutic value of the *MYC*/*NDUFV2* energy metabolism axis, a total of 77 overlapping compounds were identified from the intersection of gastric cancer drug sensitivity data (CTD and GDSC database). The result revealed 77 overlapping compounds, and the analysis ultimately focused on three core drugs: vinblastine, cisplatin, and camptothecin (CPT) (Figure 6A). By comparing the predicted IC50 values of these three drugs between high-risk and low-risk GC patients stratified by the MYC_V1 risk model, we found that the high-risk group exhibited significantly higher drug sensitivity (*p* < 0.001) (Figure 6B, upper panel). Among these, vinblastine showed the strongest theoretical binding affinity. However, based on the clinical relevance (CPT derivatives such as irinotecan are widely used in gastric cancer treatment), we selected CPT for subsequent in vitro validation. The molecular docking results further demonstrated strong binding potential between the three drugs and the NDUFV2 protein (PDB: 5XTB), with binding energies of −10.4 kcal/mol for vinblastine, −1.5 kcal/mol for cisplatin, and −7.9 kcal/mol for CPT (Figure 6B, lower panel). Cell viability assays showed that CPT treatment alone significantly inhibited cell proliferation, and *NDUFV2* knockdown exhibited a similar effect. Notably, the combination treatment group demonstrated the strongest proliferation inhibition across all tested concentrations and the IC50 of CPT in HGC-27 cells was 49 µM (Figure 6C). Therefore, 49 µM CPT was used for subsequent experiments. To validate the functional interaction between CPT and *NDUFV2*, we established four experimental groups in HGC-27 cells: a control group (si-NC), an *NDUFV2* knockdown group (si3-*NDUFV2*), a CPT-alone treatment group, and a combination treatment group (si3-*NDUFV2* + CPT). The qRT-PCR and Western blot results confirmed the effective suppression of *NDUFV2* expression in both the si3-*NDUFV2* group and the combination group (Figure 6D–F). Wound-healing assay results further indicated that the combination treatment was more effective in suppressing wound closure than either treatment alone (Figure 6G,H). These findings demonstrate that targeting the *MYC*/*NDUFV2* energy metabolism axis enhances the sensitivity of GC cells to camptothecin. Since NDUFV2 is a core subunit of mitochondrial Complex I and plays a fundamental role in cellular energy metabolism, we next investigated whether NDUFV2 knockdown affects ATP production (Figure 6I). NDUFV2 knockdown significantly reduced ATP levels in GC cells. This reduction in ATP production likely compromises the energy supply required for energy-demanding cellular processes such as proliferation and migration. These results suggest that targeting the MYC_V1/NDUFV2 axis combined with CPT treatment synergistically disrupts energy metabolism in gastric cancer cells.

## 3. Discussion

GC remains one of the most challenging malignancies worldwide, with its high mortality rate closely linked to the limited treatment options and drug resistance in advanced stages [6,39,40,41]. Therefore, exploring novel prognostic biomarkers and effective therapeutic targets is of paramount clinical importance. Through integrated bioinformatic analysis and experimental validation, this study systematically investigated the role of MYC_V1-related genes in energy metabolic reprogramming in GC and revealed the function and clinical significance of *NDUFV2* as a key effector molecule. This study first identified MYC_V1 as the primary risk factor affecting the overall survival of GC patients through cancer hallmark analysis. The prognostic risk model constructed based on MYC_V1-related genes demonstrated good predictive performance in both the training and validation cohorts, and the RS was confirmed as an independent prognostic indicator. These findings indicate that MYC_V1-driven biological processes play a central role in GC progression, and targeting this pathway may hold significant therapeutic value. Further investigation focused on *NDUFV2*, a key gene identified by the model and a core subunit of mitochondrial complex I that plays a fundamental role in cellular energy metabolism [42,43,44,45]. Our results revealed that *NDUFV2* was significantly overexpressed in GC tissues (at both mRNA and protein levels) and cell lines, and its high expression is associated with poor prognosis. This finding elevates *NDUFV2* from its traditional role as a metabolic enzyme to a potential oncogene in GC. To validate its function, we successfully knocked down *NDUFV2* expression in HGC-27 cells. Loss-of-function experiments demonstrated that *NDUFV2* knockdown significantly inhibited the proliferative and migratory capacities of GC cells. Furthermore, NDUFV2 knockdown significantly reduced ATP production in gastric cancer cells, indicating that it promotes tumor progression by regulating energy metabolism. This strongly suggests that *NDUFV2*, by regulating the cellular energy metabolic state, provides the necessary energy support for the malignant phenotypes of GC cells (such as rapid proliferation and invasion), thereby promoting GC progression.

The tumor immune microenvironment plays a critical role in GC progression. Previous studies have demonstrated that metabolic reprogramming influences immune cell infiltration in various cancers. Furthermore, TME analysis revealed significant differences in immune cell infiltration patterns between high-risk and low-risk patient groups, particularly in Macrophages M0 and NK cells. This implies that the MYC_V1/*NDUFV2* axis may indirectly influence disease progression by modulating the tumor immune microenvironment, providing a new direction for subsequent research.

Targeting metabolic dependencies has emerged as a promising therapeutic strategy in oncology. Several studies have shown that combining metabolic inhibitors with conventional chemotherapy enhances antitumor efficacy. Importantly, this study extended beyond prognostic prediction to explore the therapeutic targeting of this axis. High-throughput drug screening and molecular docking identified several compounds, including CPT, with a potential affinity for *NDUFV2*. Notably, patients in the high-risk group exhibited higher predicted sensitivity to CPT. Most significantly, in vitro experiments demonstrated that *NDUFV2* knockdown synergistically enhanced the inhibitory effects of CPT on GC cell proliferation and migration. This compelling evidence suggests that targeting the MYC_V1/*NDUFV2* axis, particularly through agents like CPT, may represent a viable strategy, especially for high-risk GC patients.

The study acknowledges several limitations that suggest directions for future research. Firstly, the precise downstream molecular mechanisms by which *NDUFV2* regulates the mitochondrial function and energy flux in GC cells remain to be fully elucidated. Due to experimental constraints, we used only one siRNA sequence for NDUFV2 knockdown and did not validate the phenotypes with additional independent siRNAs or perform rescue experiments to confirm the specificity of the observed functions. Secondly, the causal relationship and specific pathways through which the MYC_V1/*NDUFV2* axis influences the tumor immune landscape require more in-depth experimental exploration. Thirdly, the wound-healing assays were performed without proliferation inhibitors, so the observed wound closure may partly reflect cell proliferation. We plan to address these limitations in future studies with more rigorous experimental designs. Finally, the in vivo efficacy of CPT (and similar agents) in high-RS GC models and the detailed nature of its interaction with *NDUFV2* necessitate further validation in preclinical animal studies and, ultimately, clinical trials.

In summary, this study systematically elucidated the prognostic value and oncogenic function of MYC_V1-related genes, particularly *NDUFV2*, in GC. *NDUFV2* drives disease progression by promoting the proliferation and migration of GC cells, and emerges as a promising prognostic biomarker and potential therapeutic target. Our research provides new perspectives for understanding metabolic reprogramming in GC and lays a theoretical foundation for the development of precise therapeutic strategies targeting the MYC_V1/*NDUFV2* axis. Of note, although vinblastine demonstrated a stronger binding affinity to NDUFV2 in molecular docking, its role in gastric cancer remains largely unexplored. Future studies may investigate vinblastine as a potential lead compound targeting the MYC_V1/NDUFV2 axis. The main purpose of this study was to construct a prognostic risk model and preliminarily validate the function of NDUFV2. In-depth mechanistic studies will be pursued in future work.

## 4. Materials and Methods

### 4.1. Data Source and Data Processing

RNA-seq expression profiles and clinical data of 384 TCGA-STAD patients and 36 normal samples were downloaded and collected from The Cancer Genome Atlas (TCGA, https://portal.gdc.cancer.gov/) [46], and 432 GC samples were downloaded from the GSE84437 dataset in the Gene Expression Comprehensive Database (GEO, http://www.ncbi.nlm.nih.gov/geo/) [47]. We also acquired the data of 174 normal tissues from Genotype-Tissue Expression (GTEx, https://gtexportal.org/home/) [48]. Batch standardization was performed for the above data using the combat function in “sva” package in R software (version 4.2.1) [49]. Samples from patients lacking critical clinicopathological or survival information were excluded from analysis.

### 4.2. MYC_V1-Related RS, Biomarker Selection, and Signatures

To identify differences in biological functions in GC patients, the performances of cancer hallmarks in the TCGA cohort were estimated by a single-sample gene set enrichment analysis (ssGSEA) algorithm (R package “gsva”) [50] based on RNA-seq expression profiles and hallmark gene signatures from the Molecular Signatures Database (MSigDB) [51]. The significance of different cancer hallmarks in GC was assessed by a univariate Cox proportional hazards (Cox-PH) regression model. Additionally, we further acquired the most robust prognostic markers by a least absolute shrinkage and selection operator (LASSO) Cox regression model [52].

### 4.3. Validation of the Prognostic RS Model and Independent Prognostic Analysis

The GEO dataset served as an external validation cohort. Patients were stratified into high- and low-risk groups based on the median RS. Kaplan–Meier survival analysis was performed using the R “survival” package, and model accuracy was evaluated with time-dependent ROC curves [53]. Univariate and multivariate Cox regression analyses were applied to identify whether the RS was an independent prognostic factor for GC patients. A nomogram integrating the RS and clinical features was developed to predict patient prognosis [54], and its predictive accuracy was validated with calibration curves.

### 4.4. Immune Cell Infiltration Analysis

To evaluate variations in microenvironment characteristics across distinct risk subgroups, the “estimate” R package was utilized to analyze immunological and stromal components within the TME of each GC sample [55]. Utilizing the “CIBERSORT” R package, we extracted and quantified the relative percentages of 22 distinct infiltrating immune cell types present in each GC sample [56].

### 4.5. Cell Lines and Cell Culture

GC cell lines (AGS, HGC-27) and the human normal gastric mucosal cell line GES-1 were purchased from the American Type Culture Collection (ATCC; Manassas, VA, USA). The cells were cultured in Roswell Park Memorial Institute 1640 Medium (RPMI 1640 Medium; Servicebio, Wuhan, China) supplemented with 10% fetal bovine serum (FBS; Servicebio) and a 1% penicillin/streptomycin mixture in a humidified atmosphere with 5% CO_2_ at 37 °C.

### 4.6. Cell Transfection

Small-interfering RNAs (siRNAs) targeting *NDUFV2* (si*NDUFV2*#1, si*NDUFV2*#2, and si*NDUFV2*#3) and a non-targeting scrambled siRNA (negative control, siCtrl) were synthesized by Ribobio Co., Ltd. (Guangzhou, China). The target sequences were as follows: si*NDUFV2*#1: 5′-GAGAGTATATGAAGTAGCA-3′; si*NDUFV2*#2: 5′-GTGGACGCTTCTCTTGTGA-3′; and si*NDUFV2*#3: 5′-GGGAGACTACACCTGACAA-3′. AGS and HGC-27 cells were seeded into 6-well plates and cultured until reaching 60–80% confluence. Transfection was performed using riboFECT™ CP Transfection Reagent (Ribobio, Guangzhou, China; cat. no. SIGS0007222-4) at a final siRNA concentration of 50 nM, following the manufacturer’s instructions. Cells were harvested for subsequent experiments 48 h after transfection.

### 4.7. Quantitative Real-Time PCR (qRT-PCR)

Total RNA was isolated from GES-1, AGS, and HGC-27 cells using TRIzol^®^ reagent (Servicebio, China). The concentration of RNA was assessed by Nanodrop 2000/2000C spectrophotometry (Thermo Fisher Scientific, Waltham, MA, USA). Subsequently, high-quality cDNA was synthesized with SuperScript first-strand synthesis system (Thermo Fisher Scientific, Waltham, MA, USA) according to the manufacturer’s instructions. Then, qRT-PCR was performed by the 2^−ΔΔCt^ method using the AceQ qPCR SYBR green master mix (Vazyme, Nanjing, China). The primers used in qRT-PCR assays are listed in Table S1.

### 4.8. Western Blot Analysis

To validate NDUFV2 knockdown efficiency at the protein level, Western blot analysis was performed. Total protein was extracted from HGC-27 cells transfected with si-NC or si3-*NDUFV2,* or treated with CPT using RIPA lysis buffer. Following protein quantification using a BCA assay, equal protein amounts were separated by SDS-PAGE and transferred to PVDF membranes. The membranes were blocked with 5% non-fat milk and then incubated overnight at 4 °C with specific primary antibodies against NDUFV2 (dilution 1:10,000, Proteintech Company, Chicago, IL, USA) and β-actin (dilution 1:5000, Proteintech Company; used as an internal control). Following incubation with HRP-conjugated secondary antibodies, protein bands were visualized using an enhanced chemiluminescence substrate and a chemiluminescence imaging system. Band intensities were quantified with ImageJ software (version 1.54), and *NDUFV2* expression levels were normalized to β-actin.

### 4.9. Wound-Healing Assay

HGC-27 cells with or without *NDUFV2* knockdown were seeded into 6-well plates. After 24 h of culture, the complete growth medium was replaced with medium containing reduced serum concentration. Then, we scratched a wound across the cell layer using a 200 μL sterile pipette tip. Cell debris were slightly rinsed with serum-free medium two to three times. Reference points were marked on the bottom of each well with a marker pen, and images were captured at identical reference points at 0 and 24 h to ensure wound closure was evaluated in the same location. Images were captured at 0 and 24 h by a fluorescence microscope (10 × magnification, OLYMPUS, Tokyo, Japan). Wound closure rate of each group was calculated based on the migration distance.

### 4.10. CCK-8 Assay

HGC-27 cells were seeded into a 96-well plate with a cell density of 2000 cells per well and further cultured with *NDUFV2* knockdown or not at 37 °C. Then, 10 µL CCK-8 solution (Servicebio, China) was added into the wells and incubated for 4 h, and the absorbance at 450 nm was measured with a microplate reader (Tecan infinite, Männedorf, Switzerland). Each assay was conducted in triplicate and the cell growth curve was created [57].

### 4.11. Drug Sensitivity Analysis

To evaluate the sensitivity of individual GC patients to chemotherapeutic agents, we utilized the Genomics of Drug Sensitivity in Cancer (GDSC, https://www.cancerrxgene.org/) database [58] and the Comparative Toxicogenomics Database (https://ctdbase.org), and the IC50 was used to quantify using the “oncoPredict” package of R [59].

### 4.12. Molecular Docking

Based on previous network of pharmacology findings, key active compounds (e.g., vinblastine, CPT, and cisplatin) and core target proteins (e.g., 5XTB) were selected as ligands and receptors, respectively. Ligand 3D structures were obtained from PubChem, and receptor crystal structures were downloaded from RCSB PDB. Molecular docking was performed using CB-Dock2 with the Vina algorithm. Binding energy (<−5.0 kcal/mol for good binding, and <−7.0 kcal/mol for strong binding) and RMSD (<2.0 Å) were used as evaluation criteria. The optimal binding conformation was visualized and analyzed for interactions and binding sites.

### 4.13. ATP Production Assay

Intracellular ATP levels were measured using an ATP assay kit (Beyotime, Shanghai, China; cat. no. S0026) according to the manufacturer’s instructions. Briefly, HGC-27 cells were seeded into 6-well plates and transfected with si-NC or si-NDUFV2 for 24 h. After transfection, cells were treated with CPT (49 µM) or vehicle control (DMSO) for an additional 24 h. Cells were then lysed with ATP lysis buffer, and the lysates were centrifuged at 12,000× *g* for 5 min at 4 °C. The supernatant was collected, and ATP concentration was determined by measuring luminescence using a microplate reader (Tecan infinite). Protein concentration was measured using a BCA assay kit, and ATP levels were normalized to total protein content. Each experiment was performed in triplicate and repeated three times independently.

### 4.14. Statistical Analysis

The differences between the groups were assessed using *t*-tests. The results were plotted and analyzed using R software version 4.1.3. Data visualization was performed using GraphPad Prism 9.0, with *p* < 0.05 indicating statistical significance.

## 5. Conclusions

In conclusion, this study systematically elucidated the prognostic value and oncogenic function of MYC_V1-related genes in GC. The prognostic risk model constructed based on eight MYC_V1-related genes was successfully developed and validated across independent cohorts. *NDUFV2* emerged as a key gene within this prognostic signature. Functional experiments confirmed that *NDUFV2* drives GC progression by promoting tumor cell proliferation and migration. These findings highlight *NDUFV2* as a critical mediator of MYC_V1-driven metabolic reprogramming. Collectively, NDUFV2 represents a promising prognostic biomarker for GC patients and a potential therapeutic target for clinical intervention.

## Data Availability

Data for TCGA-STAD patients and normal samples were downloaded from The Cancer Genome Atlas (TCGA, https://portal.gdc.cancer.gov/). GC sample data were retrieved from the GSE84437 dataset in the Gene Expression Omnibus (GEO) database (http://www.ncbi.nlm.nih.gov/geo/). Normal tissue data were obtained from the Genotype-Tissue Expression (GTEx) project (https://gtexportal.org/home/).

## Supplementary Materials

The following supporting information can be downloaded at: https://www.mdpi.com/article/10.3390/ijms27114862/s1.

## Abbreviations

The following abbreviations are used in this manuscript:

GC	Gastric Cancer
scRNA-seq	single-cell RNA sequencing
ssGSEA	single-sample Gene Set Enrichment Analysis
RS	Risk Score
OS	Overall Survival
CPT	Camptothecin
LASSO	Least Absolute Shrinkage and Selection Operator
KM	Kaplan–Meier
ROC	Receiver Operating Characteristic
IHC	Immunohistochemistry
C-index	Concordance index
GEO	Gene Expression Omnibus
TME	Tumor Microenvironment
RNA-seq	RNA sequencing
GDSC	Genomics of Drug Sensitivity in Cancer
