# Supplementary material for: MYC_V1-Related Genes Affect Gastric Cancer Proliferation by Regulating Energy Metabolism and Analysis of Therapeutic Targets
Source: Int J Mol Sci. 2026 May 28;27(11):4862. doi: 10.3390/ijms27114862 (PMC13256221; doi:10.3390/ijms27114862)
Supplement: Supplementary file 1 [file ijms-27-04862-s001.zip › Supplementary figures_01.pdf]

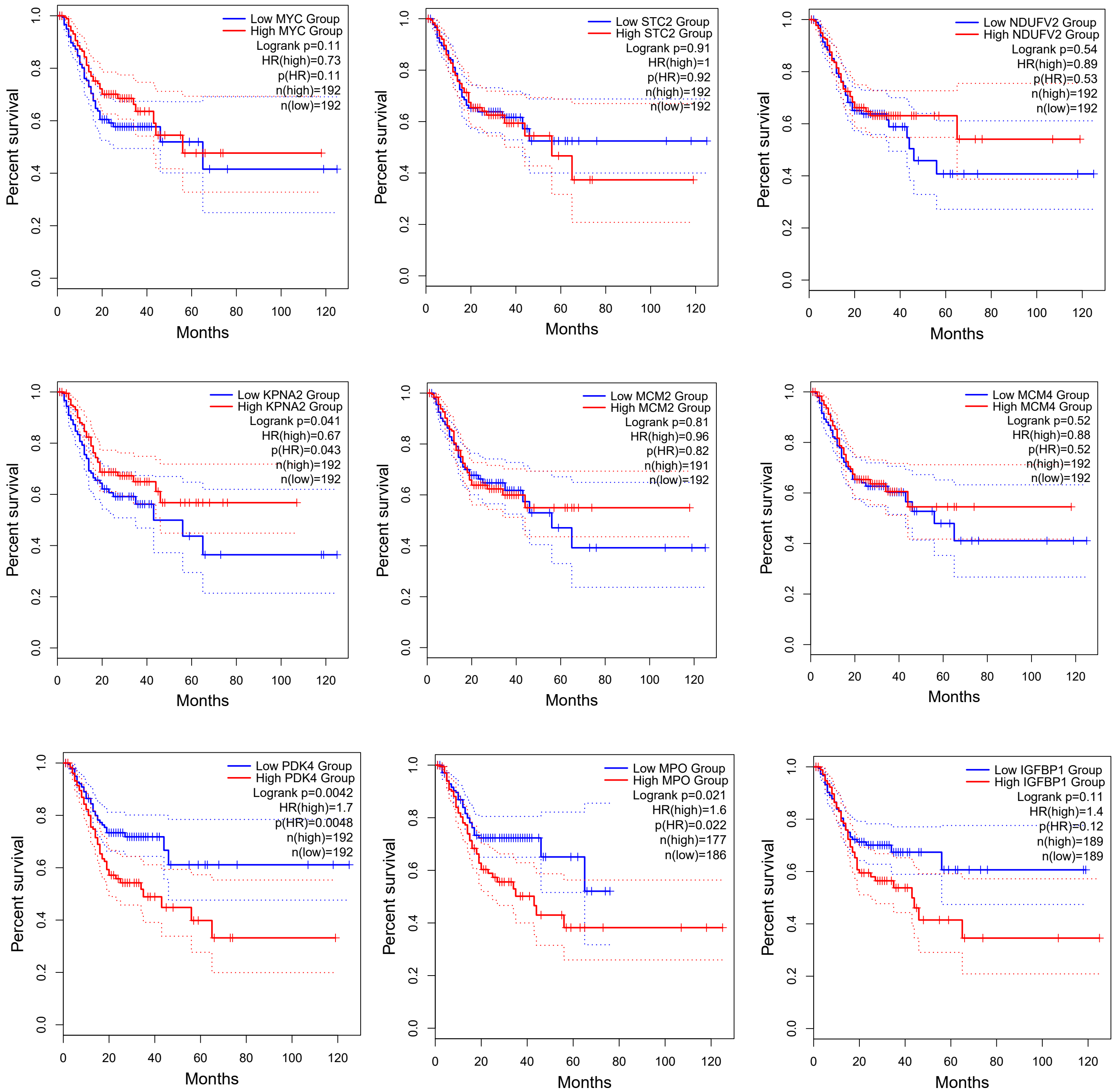

Supplementary figure S1. Association between individual prognostic gene expression levels and disease-free survival in GC patients. Disease-free survival curves for the MYC and 8 key genes in the prognostic model stratified by high and low expression groups.
