# Supplementary material for: MYC_V1-Related Genes Affect Gastric Cancer Proliferation by Regulating Energy Metabolism and Analysis of Therapeutic Targets
Source: Int J Mol Sci. 2026 May 28;27(11):4862. doi: 10.3390/ijms27114862 (PMC13256221; doi:10.3390/ijms27114862)
Supplement: Supplementary file 1 [file ijms-27-04862-s001.zip › Supplementary figures_02.pdf]

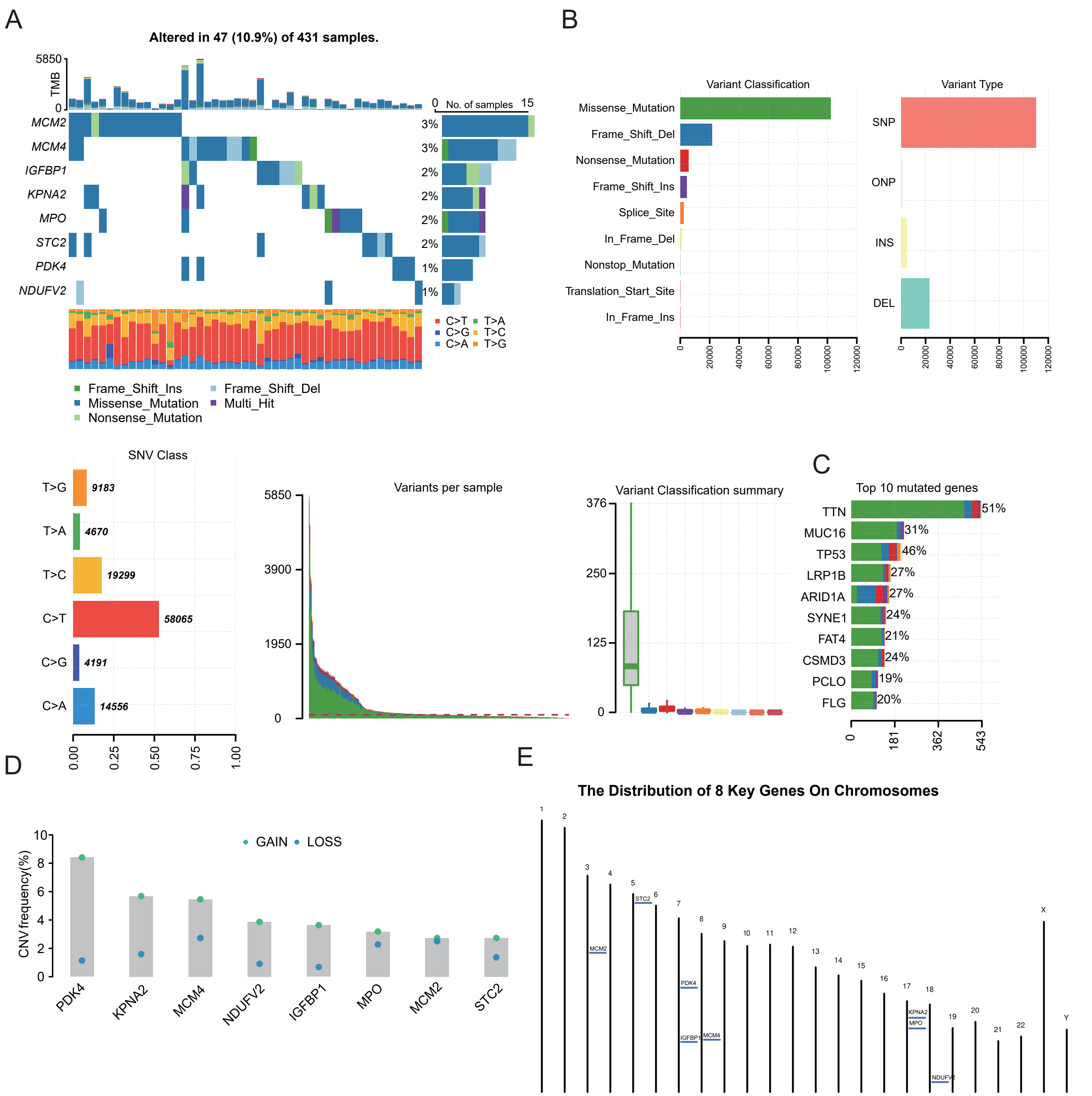

Supplementary figure S2. Genetic landscape of the MYC_V1-related prognostic signature in GC. (A) Mutation spectrum of the key genes in the TCGA-GC cohort. (B) Overview of mutation patterns including variant classification, type, SNV class, and TMB. (C) Summary of the top 10 mutated genes in GC. (D) Copy number variation (CNV) frequency of the 8 key genes. (E) The distribution of 8 key genes on chromosomes.
