# Supplementary material for: MYC_V1-Related Genes Affect Gastric Cancer Proliferation by Regulating Energy Metabolism and Analysis of Therapeutic Targets
Source: Int J Mol Sci. 2026 May 28;27(11):4862. doi: 10.3390/ijms27114862 (PMC13256221; doi:10.3390/ijms27114862)
Supplement: Supplementary file 1 [file ijms-27-04862-s001.zip › Supplementary figures_04.pdf]

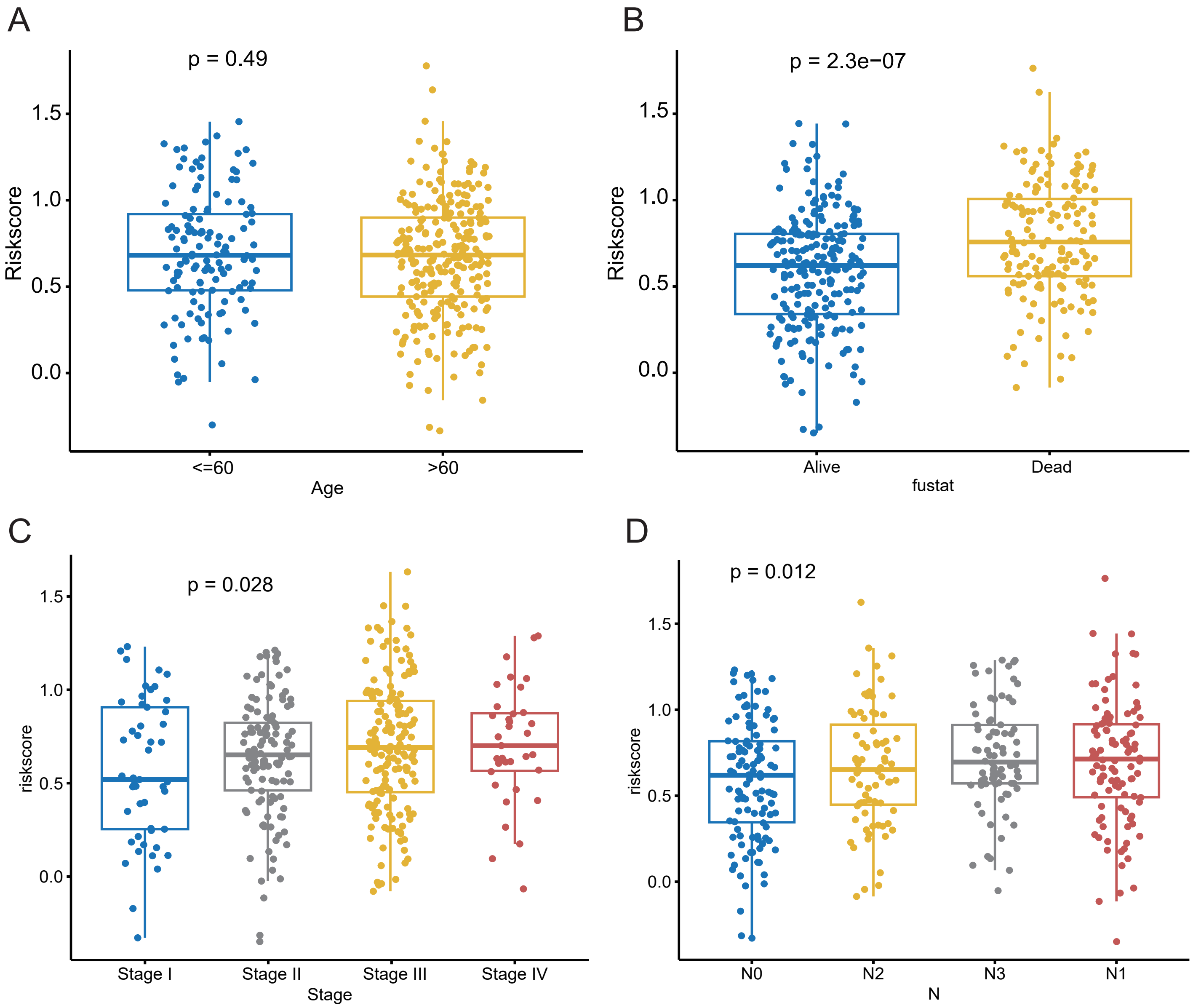

Supplementary figure S4. Correlation analysis between risk score and clinical characteristics of GC. (A) Risk score between patients of different age groups. (B) Bar chart showing the correlation between the patient's survival status and risk score. (C) The relationship between risk scores of patients with different TNM stages. (D) The correlation of risk scores among patients with different degrees of lymph node metastasis.
