# Supplementary material for: MYC_V1-Related Genes Affect Gastric Cancer Proliferation by Regulating Energy Metabolism and Analysis of Therapeutic Targets
Source: Int J Mol Sci. 2026 May 28;27(11):4862. doi: 10.3390/ijms27114862 (PMC13256221; doi:10.3390/ijms27114862)
Supplement: Supplementary file 1 [file ijms-27-04862-s001.zip › Supplementary figures_05.pdf]

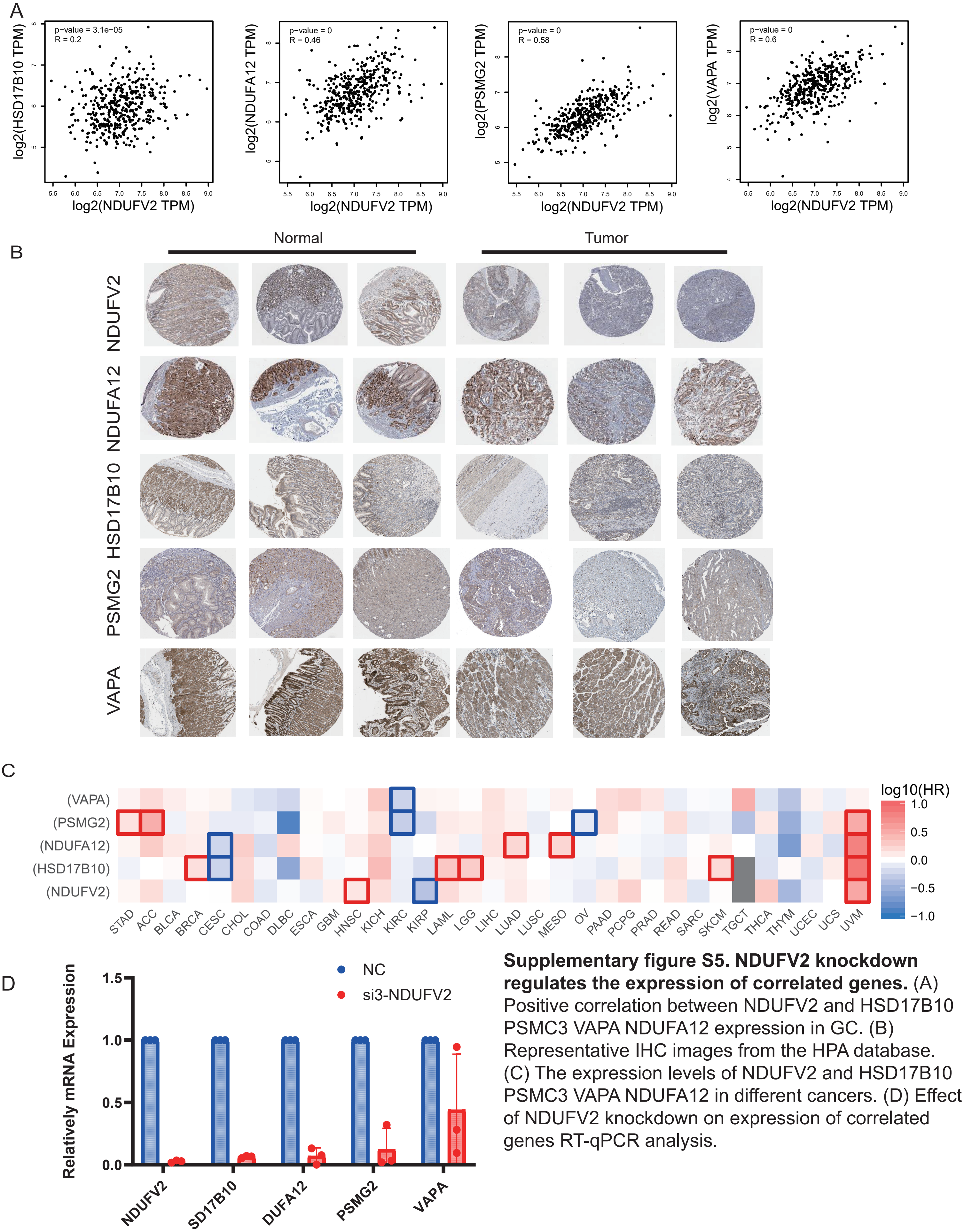

Supplementary figure S5. NDUFV2 knockdown regulates the expression of correlated genes. (A) Positive correlation between NDUFV2 and HSD17B10 PSMC3 VAPA NDUFA12 expression in GC. (B) Representative IHC images from the HPA database. (C) The expression levels of NDUFV2 and HSD17B10 PSMC3 VAPA NDUFA12 in different cancers. (D) Effect of NDUFV2 knockdown on expression of correlated genes RT-qPCR analysis.
