# Supplementary material for: MYC_V1-Related Genes Affect Gastric Cancer Proliferation by Regulating Energy Metabolism and Analysis of Therapeutic Targets
Source: Int J Mol Sci. 2026 May 28;27(11):4862. doi: 10.3390/ijms27114862 (PMC13256221; doi:10.3390/ijms27114862)
Supplement: Supplementary file 1 [file ijms-27-04862-s001.zip › Table S1.pdf]

Table S1 The list of primer sequences used in our study

Number	Name	Primer (5' to 3')
1	GAPDH-F	TGACTTCAACAGCGACACCCA
2	GAPDH-R	CACCCTGTTGCTGTAGCCAAA
3	NDUFV2-F	GGTTGGGGAGACTACACCTGA
4	NDUFV2-R	CTTGGCCCTGGTTTTGGGAT
5	NDUFVA12-F	GGTCTCCGAGGCTATCTACGG
6	NDUFVA12-R	GGAGGCACCATGCTTCCATC
7	MYC-F	CACACCCACAATTCAGGAAGAG
8	MYC-R	GACGTGCTACAAGGTGGCA
9	VAPA-F	ACCTAGCAAAGCTGTTCCACT
10	VAPA-R	CTCAGGTGTCGGTTTTCTTCTG
11	HSD17B10-F	CTGGTGAGATGGGCCAGAATG
12	HSD17B10-R	CCAACCTGACCCTCGAAGG
13	PSMG2-F	ACCGATTGTCTTGTGCCAATG
14	PSMG2-R	AGGCAATGAATACACTTCAGCAT
